# Keystone Veterinary Medical Association

**Published:** 1892-02

**Authors:** W. S. Kooker

**Affiliations:** Secretary


*Keystone Veterinary Medical Association.*—The regular monthly meeting of the Keystone Veterinary Medical Association met at the College of Physicians, Philadelphia, January 9th, at 8 P. M, the President, Dr. Hoskins, in the chair. Drs. Goentner, Rayner, Kooker, Lintz, Webster and Evas answered to roll-call. The minutes of previous meeting were read and approved. The President appointed Drs. Goentner, Rayner, Evas and Lintz to act in place of absent trustees. The application of Dr. H. B. McDowell was reported favorably by the Board of Trustees. Reports accepted, and Dr. McDowell was duly elected a member. The essayist of the evening not being able, on account of a sprained ankle, to be present, Dr. W. Horace Hoskins reported a case and submitted a specimen of diseased colen with an interesting history of the same; he also reported several cases of azoturia. His treatment consisted of potassii acetate, plenty of water and hot clothes on the affected muscles. Several of the cases were one-legged, two had both posterior limbs affected, and died. He was opposed to the use of aloes in the treatment, which in his experience had proved to be harmful. Dr. Kooker reported seven cases due to the New Year's holidays, six were one-legged and in the other both hind limbs were affected and it was down. Treated with aloes purge and potash nit., with no external treatment, and all recovered. The one with both hind limbs affected is now going about in good condition. Dr. Lintz asked several questions and made some pertinent remarks on the treatment of the disease.

There was a motion made and seconded that the date of meeting be changed from the first Saturday of the month to the first Friday, in order to accomodate several members who can not attend on Saturday. The motion was carried. On motion adjourned.

W. S. KOOKER, *Secretary.*

